# *Candida Albicans* Virulence Factors and Its Pathogenicity

**DOI:** 10.3390/microorganisms9040704

**Published:** 2021-03-29

**Authors:** Mariana Henriques, Sónia Silva

**Affiliations:** CEB-Centre of Biological Engineering, University of Minho, 4710-057 Braga, Portugal; soniasilva@deb.uminho.pt

*Candida albicans* lives as commensal on the skin and mucosal surfaces of the genital, intestinal, vaginal, urinary, and oral tracts of 80% of healthy individuals. An imbalance between the host immunity and this opportunistic fungus may trigger mucosal infections followed by dissemination via the bloodstream and infection of the internal organs. *Candida albicans* is considered the most common opportunistic pathogenic fungus in humans and a causative agent of 60% of mucosal infections and 40% of candidemia cases [1,2]. Several virulence factors are known to be responsible for *C. albicans* infections, such as adherence to host and abiotic medical surfaces, biofilm formation as well as secretion of hydrolytic enzymes. Moreover, *C. albicans* resistance to traditional antimicrobial agents, especially azoles, is well known, especially when *Candida* cells are in biofilm form.

This Special Issue covers different aspects related to *C. albicans* pathogenicity, virulence factors, the mechanisms of antifungal resistance and the molecular pathways of host interactions. The review by Ciurea et al. [3] presents the virulence factors of the most important *Candida* species, namely *C. albicans,* contributing to a better understanding of the onset of candidiasis and raising awareness of the overly complex interspecies interactions that can change the outcome of the disease. The article by Yoo et al. [4] provides a comprehensive review about the association between *C. albicans* and the cases of persistent or refractory root canal infections. It also points out the importance of alternative intracanal medicaments such as chlorhexidine gel or human beta defensin-3 (HBD3), Ca-Si-based obturating materials, and microsurgical procedures. Zambom et al. [5] presented a review on the promising alternatives of the use of antifungal peptides (AFPs) from the Histatin family (like histatin-5) and nanoparticles (NPs) for the treatment of candidiasis. The article reveals how nanotechnology can allow the application of AFPs and NPs for the treatment of *Candida* infections. Rosati et al. [6] provided an overview of the current understanding of the host immune response in vulvovaginal candidiasis (VVC) pathogenesis and suggests that a tightly regulated fungus–host–microbiota interplay might exert a protective role against recurrent *Candida* infections. The review by Satala et al. [7] describes the importance of *C. albicans* cell wall proteins not only as a protective envelope but also as a point of contact with the human host, providing a dynamic response to the constantly changing environmental infection niches. The sixth review article in the Special Issue (Costa-de-Oliveira et al. [8]) describes the main factors that are involved in antifungal resistance and tolerance in patients with *C. albicans* bloodstream infections. Azoles are widely used drugs in the treatment of candidiasis, which target the lanosterol 14α-demethylase (Erg11p) encoded by the *ERG11* gene, therefore the data of Suchodolski et al. [9] showed that targeted gene disruption of *ERG11* can result in resistance to ergosterol-dependent drugs (azoles and amphotericin B). They suggested that this new insight into intracellular processes under Erg11p inhibition may lead to a better understanding of the indirect effects of azoles on *C. albicans* cells and the development of new treatment strategies for resistant infections. In addition, the same authors [10] proposed a new method for the detection of cell membrane depolarization/permeabilization in *C. albicans* using the potentiometric zwitterionic dye di-4-ANEPPS. The data presented by Caldara et al. [11] suggest that nortriptyline can be considered a “new” antimicrobial drug with great potential for application in *in vivo C. albicans* infection models. Therapies targeted to fungal biofilms, mainly against the matrix, and therapies that do not induce microbial resistance are relevant. N-acetylcysteine (NAC), a mucolytic agent, has shown antimicrobial action. Nunes et al. [12] evaluated the effect of NAC against fluconazole-susceptible and -resistant *C. albicans* and the results revealed that high concentrations of NAC had similar fungistatic effects against both strains, while a low concentration showed the opposite result. Ubiquinones (UQ) are intrinsic lipid components of many membranes and Pathirana et al. [13] provided specific exam of Ubiquinones (UQ) and proved the significance of UQ side chains in farnesol production and resistance quite apart from being an electron carrier in the respiratory chain of *C. albicans* cells. MAP kinase (MAPK) signal transduction pathways facilitate the sensing and adaptation of *C. albicans* cells to external stimuli and control the expression of key virulence factors such as the yeast-to-hypha transition, the biogenesis of the cell wall, and the interaction with the host. Correia et al. [14] demonstrated that the four MAPK pathways play distinct roles in adhesion, epithelial damage, invasion, and cell wall remodelling that may contribute to the pathogenicity of *C. albicans*. The behaviour of *C. albicans* on simulated human body fluids (artificial saliva and urine) at different values of pH (pH 5.8 and 7) was analysed by Barbosa et al. [15]. The authors demonstrated that *C. albicans* presents high plasticity and adaptability to different human body fluids, namely saliva and urine. Interestingly, Tseng et al. [16] showed that unlike *C. albicans*, the *C. tropicalis ROB1* deletion strain did not cause a significant reduction in biofilm formation, suggesting that the biofilm regulatory circuits of the two species are divergent.

Overall, the 14 papers published in this Special Issue nicely illustrate why the *C. albicans* continues to be one of the most common opportunistic pathogenic fungi in humans and highlights the importance of focusing research on understanding the mechanisms of antifungal resistance and its pathogenicity.
